# Circulating bile acids and HOMA-IR: cross-sectional results from the RoCAV population-based study

**DOI:** 10.3389/fendo.2025.1656942

**Published:** 2025-10-15

**Authors:** Sarah Grossi, Emanuele Maria Giusti, Giovanni Veronesi, Simone Delaiti, Andrea Mancini, Laura Migliaccio, Selene Genova, Simona Costanzo, Kieran Michael Tuohy, Marco Mario Ferrario, Francesco Gianfagna

**Affiliations:** ^1^ Research Center in Epidemiology and Preventive Medicine (EPIMED), Department of Medicine and Surgery, University of Insubria, Varese, Italy; ^2^ Research and Innovation Centre, Fondazione Edmund Mach, San Michele all’Adige, Italy; ^3^ Dipartimento di Prevenzione, Azienda Sanitaria Locale Asl Novara, Novara, Italy; ^4^ Research Unit of Epidemiology and Prevention, IRCCS Neuromed, Pozzilli, Italy; ^5^ School of Food Science & Nutrition, University of Leeds, Leeds, United Kingdom; ^6^ Health Directorate and Socio-Health Directorate, ASST Sette Laghi, Varese, Italy

**Keywords:** bile acids, fibroblast growth factor 19, HOMA-IR, microbiota biomarkers, type 2diabetes

## Abstract

**Background:**

Circulating bile acids (cBAs) function as signaling molecules that activate the farnesoid X receptor (FXR), promoting the secretion of fibroblast growth factor 19 (FGF-19), a gut-derived hormone involved in bile acid (BA) synthesis, glucose metabolism, and insulin sensitivity. However, the relationship between fasting cBAs, FGF-19, and insulin resistance—as estimated by HOMA-IR—remains unclear. This study explored these associations in an elderly population from Northern Italy.

**Material and methods:**

We examined a subsample of 1080 subjects (aged 60–75 years, 1:1 male-to-female ratio) from the RoCAV population-based study (2013–2016). Fasting blood samples were analyzed for 33 cBAs using UHPLC-MS/MS, of which 23 met quality criteria. FGF-19 levels were also measured. After excluding individuals with missing data or fibrate therapy, 1049 participants were included. Associations between cBAs, FGF-19, and HOMA-IR were assessed via linear regression, adjusting for age, sex, BMI, diet, alcohol intake, hypertension, and dyslipidemia. ROC curve analysis evaluated the ability of cBAs and FGF-19 to discriminate T2DM cases.

**Results:**

After excluding participants with missing anthropometric and clinical data or in fibrate treatment, data from 1049 subjects (mean±SD age 68.6±4.5 years, males 49.5%, T2DM 10.6%) were analysed. FGF-19 showed a positive correlation with primary cBAs (Spearman’s ρ=0.33, p<0.001). Adjusted for covariates, both primary and secondary cBAs were positively associated with HOMA-IR (β=0.07, p=5×10^-5^; β=0.9, p=4×10^-7^) while FGF-19 was not (β=-0.02, p=0.31). In the mutually-adjusted model - including primary and secondary cBAs, FGF-19, and covariates - the β coefficients for cBAs were attenuated but remained significant (primary: β=0.06, p=0.005; secondary: β=0.07, p=0.0003), and FGF-19 retained an inverse association (β=–0.05, p=0.009). When total cBAs were used in the FGF-19–adjusted model, the association with HOMA-IR was the strongest (β=0.19, p=5×10^-19^). ROC curve analysis indicated that the inclusion of primary and secondary cBAs and FGF-19 improved model discrimination for T2DM (ΔAUC=0.03, 95% Confidence Interval: 0.01-0.06; Net Reclassification Improvement=0.54; 95%CI: 0.30-0.75).

**Conclusions:**

In this elderly Italian population, primary and secondary cBAs were positively associated with insulin resistance, after adjusting for each other, whereas FGF-19 negatively. These markers may enhance T2DM risk stratification and may give insights on bile acid–glucose metabolism links.

7-dehydro-CA, 7-DehydroCholic acid; 7-keto LCA, 7-Ketolithocholic acid; BMI, Body mass index; BP, Blood pressure; CA, Cholic acid; CABALA, CirculAting Bile Acids as biomarkers of metabolic health - Linking microbiotA; CBAs, Circulating bile acids; CA-GLUC, Cholic acid-Glucoronate; CA-SULF, Cholic acid-Sulfate; CDCA, ChenoDeoxyCholic acid; CDCA-GLUC, ChenoDeoxyCholic acid-glucoronate; CI, Confidence intervals; CYP7A1, Cytochrome P450 family 7 subfamily A member 1; CYP8B1, Cytochrome P450 family 8 subfamily B member 1; DCA, DeoxyCholic acid; DCA-GLUC, DeoxyCholic acid-glucoronate; Dehydro-CA, DehydroCholic acid; ELISA, Enzyme-linked immunosorbent assay; EPIC, European Prospective Investigation on Cancer and Nutrition; FGF-19, Fibroblast Growth Factor 19; FXR, Farnesoid X receptor; GCA, Glycocholic acid; GCDCA, GlycoChenoDeoxycholic acid; GCDCA-SULF, GlycoChenoDeoxycholic acid-sulfate; GDCA, GlycoDeoxycholic acid; GDCA-SULF, GlycoDeoxycholic acid-sulfate; GHCA, GlycoHyocholic acid; GHDCA, GlycohyoDecoxycholic acid; GLCA, GlycoLithocholic acid; GUDCA, GlycoUrsoDeoxycholic acid; GUDCA-SULF GlycoUrsoDeoxycholic acid-sulfate; HDL, High-density lipoprotein; HOMA-IR, Homeostatic Model Assessment for Insulin Resistance index; IR, Insulin resistance; LCA, LithoCholic acid; LCA-SULF, LithoCholic acid-Sulfate; LDL, Low-density lipoprotein; LOD, Level of Definition; MedScore, Mediterranean Diet Score; NAFLD, Non-alcoholic fatty liver disease; NRI, Net Reclassification Improvement; RCT, Randomised control trials; RoCAV, Risk of Cardiovascular diseases and abdominal aortic Aneurysm in Varese; SD, Standard deviation; T2DM, Type 2 diabetes; TCA, TauroCholic acid; TCA-SULF, TauroCholic acid-sulfate; TCDCA, TautoChenoDeoxycholic acid; TDCA, TauroDeoxycholic acid; TGR5, G-protein-coupled bile acid receptor 5; THCA, TauroHyoCholic acid; THDCA, TauroHyoDeoxycholic acid; TLCA, TauroLithocholic acid; TLCA-SULF, TauroLithocholic acid-sulfate; TUDCA, TauroUrsodeoxycholic acid; UCA, UrsoCholic acid; UDCA, UrsoDeoxyCholic acid; UHPLC-MS/MS, Ultrahigh performance liquid chromatography tandem mass spectrometry method.

## 1 Introduction

Circulating bile acids (cBAs) are a group of amphoteric sterols traditionally known for their role in digestion, promoting the emulsification and absorption of lipids in the intestine (1). Primary bile acids, mainly cholic acid (CA) and chenodeoxycholic acid (CDCA), are synthesized from cholesterol in the liver and released into the small intestine in response to food intake, to aid lipid absorption and cholesterol catabolism. Within the gut, they are converted into secondary BA by the microbiota (2).

In the liver and peripheral tissues cBAs can act as signaling molecules to activated different receptor, including the farnesoid X receptor (FXR) and the G-protein-coupled bile acid receptor 5 (TGR5), to modulate effects on glucose and lipid metabolism, enhances insulin sensitivity, and maintains energy homeostasis (3, 4).

Fibroblast Growth Factor 19 (FGF-19), a hormone primarily produced in the ileum, plays several roles in response to bile acids. When BAs enter the ileum after a meal, they stimulate the release of FGF-19 into the bloodstream, which acts on the liver and other tissues via its receptor (Fibroblast Growth Factor Receptor 4). Its primary function is to regulate bile acid synthesis by downregulating cytochrome P450 family 7 subfamily A member 1 (CYP7A1) and cytochrome P450 family 8 subfamily B member 1 (CYP8B1), which convert cholesterol into bile acids. This helps prevent the accumulation of excess bile acids in the body. In this way, FGF-19 helps to maintain a balance in bile acid levels (5).

It has been reported that individuals with type 2 diabetes (T2DM) exhibit altered circulating levels of both primary and secondary bile acids compared to non-diabetic individuals (6). These changes often include an increased proportion of secondary bile acids, suggesting enhanced microbial conversion. This altered bile acid profile may impair activation of the FXR–FGF-19 axis, leading to reduced FGF-19 signaling.

FGF-19 has been increasingly studied for its role in metabolic regulation, particularly in the context of insulin resistance (IR), and appears to influence insulin sensitivity (7, 8). One of the metrics used to assess insulin resistance in clinical studies is the Homeostasis Model Assessment of Insulin Resistance (HOMA-IR), which is calculated based on fasting blood glucose and insulin levels (9). Elevated HOMA-IR values typically indicate a state of insulin resistance, a key feature of metabolic disorders such as T2DM, obesity, and non-alcoholic fatty liver disease (NAFLD) (10–12). It was described that in presence of level of FGF-19 circulating glucose levels were reduced through hepatic, central, and peripheral targeting (8), however, the mechanism by which it acts has not been fully defined.

The present study aims to investigate the association of HOMA-IR with both circulating bile acids and FGF-19. Results could be useful to delineate the specific roles of bile acids and FGF-19 in glucose metabolism and their potential as targets for diabetes treatment and prevention.

## 2 Materials and methods

### 2.1 Study population

**Figure 1:**
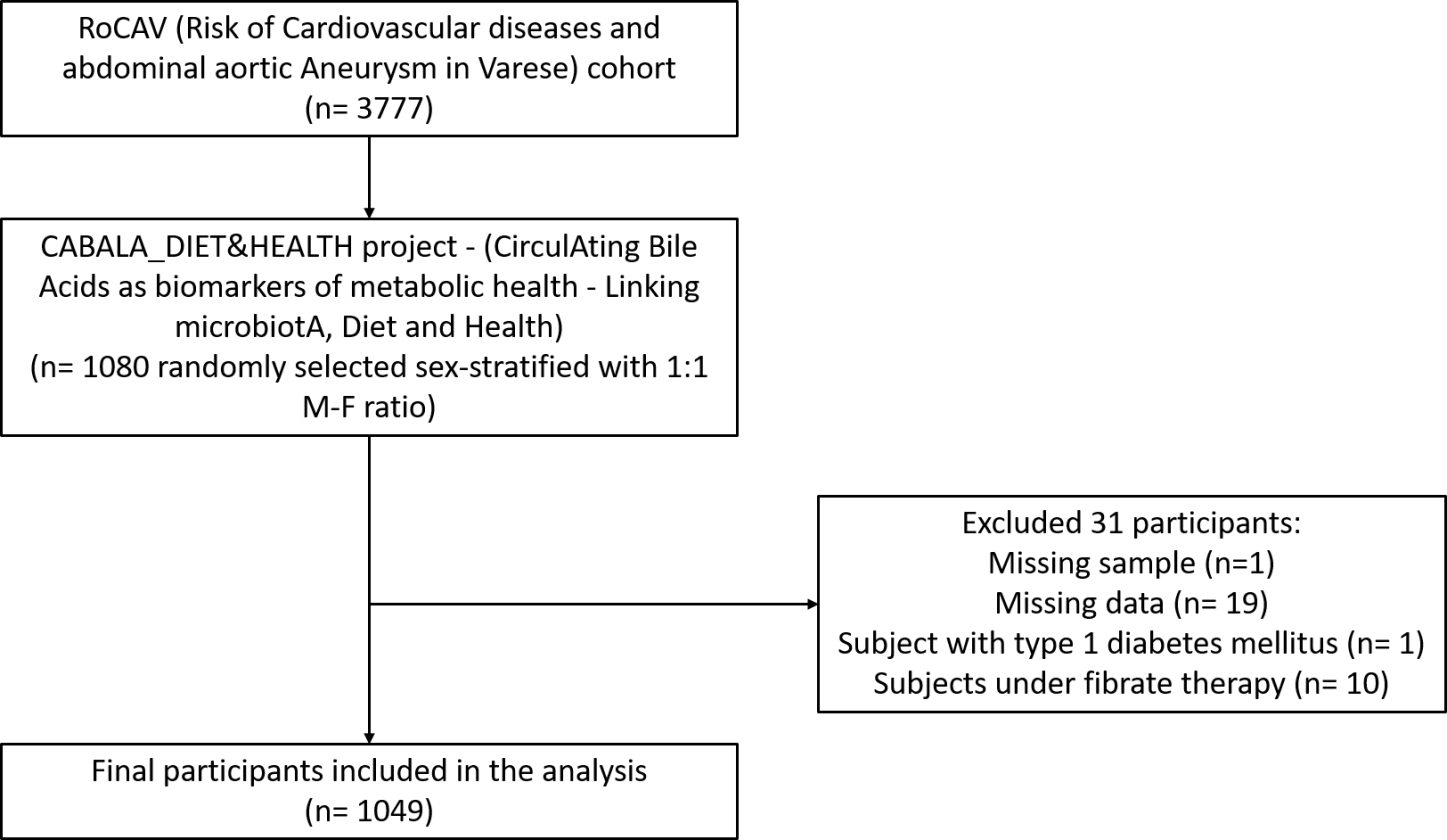
Flowchart of study population.

This work was conducted in the framework of the CABALA_DIET&HEALTH project (CirculAting Bile Acids as biomarkers of metabolic health - Linking microbiotA, Diet and Health), a subanalysis of the RoCAV (Risk of Cardiovascular diseases and abdominal aortic Aneurysm in Varese) cohort (13). The RoCAV is a population-based cohort that enrolled 3777 individuals (63.7% male, 65.5±6.7 years), randomly selected from the civil registry of Varese city between 2013 and 2016. For the CABALA project, a sex-stratified (1:1 male-female ratio) subsample of 1080 subjects aged 60 to 75 years was randomly selected. Biological samples were collected from the selected participants. Subjects with missing values in laboratory data or anthropometry, with type 1 diabetes, and under fibrate therapy were excluded, leading to a final sample of 1049 individuals (**Figure 1**).

The RoCAV study was approved by the local Varese Hospital Ethical Committee (n. 66/2011, date 8^th^ Jan 2013) and each participant signed two separate written informed consents: one for study participation and one for sample storage in the biobank.

### 2.2 Data collection

For this study, we used socio-demographic, anthropometric, clinical and pharmacological history, lifestyle and laboratory data collected during the baseline visit.

Socio-demographic, pharmacological history and lifestyle data were collected by trained operators using computerized questionnaires. Among the lifestyle data, a specific questionnaire on eating habits for the quantification of the main food groups (EPIC questionnaire) was used. The questionnaire investigates the consumption in terms of quantity and frequency of individual foods or groups of foods in the year prior to the survey (14). Nutritional variables were then derived using standard algorithms. For this study, we used total food intake (the edible part in grams/day of declared food) and alcohol consumption (alcohol from wine, beer and spirits in grams/day) (15).

Anthropometric and clinical measures were ascertained by trained physicians. Prevalent diabetes, hypertension, and hyperlipidemia were defined as dichotomous variables (Yes/No), based on the reported and verified use of specific drugs for the treatment of these disorders. Body mass index (BMI) was calculated as kg/m2. Blood pressure was measured three times following standardized protocol. All methods adhered to the standardized procedures and quality standards of the MONICA Project (16).

A blood sample were collected (overnight fasting) between 8:00 a.m. and 9:30 a.m., for laboratory analysis. Blood cell count, serum lipids and blood glucose were analyzed in the centralized hospital laboratory using commercial reagents and automatic analyzers. LDL-cholesterol was calculated using the Friedewald formula.

In the framework of CABALA project, plasma from overnight fasting blood samples stored in -80 °C were used to quantify FGF-19 and insulin using commercial ELISA kit (ELLA Technologies). Additionally, cBAs were quantified in the same samples following a validated method based on UHPLC-MS/MS (17), with 94% of the overall samples reporting values within 70-130% relative accuracy range, 8.3% mean precision, <30% coefficient of variation (18).

### 2.3 Statistical methods

A data quality assessment (available for all phenotypes collected at baseline, epimed.uninsubria.eu) was performed on dietary data, checking data completeness, plausibility (energy intake <4000 kcal/day for men, <3500 kcal/day for women), internal consistency, seasonality and external validity (19). This assessment lead to the exclusion of subjects with unreliable values before subsample selection. Data quality assessment was also performed on insulin, cBAs and FGF-19. Techniques accounting for left-censoring were applied for data falling below the limit of detection, which were imputed between Limit of Detection (LOD)/2 and LOD. Values exceeding the Linearity Range were replaced with its upper limit. We excluded from the analyses of individual cBAs those with more than 40% of imputed values. **Supplementary Table 1** presents the LOD and linearity range limits, the number of imputed values, and the means with standard deviations of the bile acid levels for the entire CABALA project sample.

Then, we described categorical variables using frequencies and percentages, numeric socio-demographic and clinical variables using means and standard deviations, and bile acids levels using geometric means at the original scale and arithmetic means and standard deviations at the log scale. Differences between study participants across T2DM status were ascertained using t-tests. Correlations among bile acids were investigated using Spearman’s correlation coefficient.

We then investigated the associations between HOMA-IR and the total, primary and secondary cBA groups, as well as FGF-19, performing multiple regression models. First, we fitted minimally-adjusted models for each cBA group and FGF-19 independently, adjusting for age, sex, alcohol consumption, and total food intake. Next, we fitted fully-adjusted models by adding BMI, hypertension, and dyslipidemia to the minimally-adjusted models (Model 2). Next, for the total, primary, and secondary cBAs, we fitted models that included the variables from Model 1 and FGF-19 levels (Model 3). Finally, we fitted a single mutually-adjusted model, which included all the variables from the fully-adjusted model (Model 2) and entered the cBA groups and FGF-19 simultaneously.

These analyses were repeated in the subsample of individuals without T2DM. In addition, the minimally-adjusted models and the FGF-adjusted models were run for each single cBA. For all these analyses, cBAs and their groupings were log-transformed and standardized and HOMA index was log-transformed. Multicollinearity was assessed using the Variance Inflation Factor. P-values for the coefficients of the cBAs, cBA groups and FGF-19 were corrected for controlling the false discovery rate using the Benjamini-Hochberg procedure (q=0.01).

Finally, to ascertain whether the cBAs are able to characterize the presence of T2DM, we compared a logistic model that included the variables of the fully-adjusted models with one that also included the cBA groups (primary and secondary) and FGF-19 by calculating the change in AUC (ΔAUC) and the continuous Net Reclassification Improvement (NRI), with 95% bootstrap confidence intervals (CI).

The analyses were performed using SAS (version 9.4, SAS Institute Inc. Cary, NC, USA) and R (version 4.4.1, CRAN Project, www.r-project.org).

## 3 Results

### 3.1 Baseline characteristics of the study population

**Table 1** reports characteristics of the 1049 subjects included in the analysis, stratified by the presence of type 2 diabetes.

**Table 1:** Socio-demographic, anthropometric and clinical features of the study sample, overall and by diabetes status (CABALA Study, N 1049).

Variables	All subjects (n=1049)	Non-T2DM (n=938)	T2DM (n=111)	p-value
Age (years), mean±SD	68.6±4.5	68.5±4.5	69.3±4.3	0.06
Female sex, n (%)	530 (50.5)	490 (52.2)	40 (36.0)	0.002
Ever smoker, n (%)	472 (45.0)	410 (43.7)	62 (55.9)	0.02
BMI (kg/m^2^), mean±SD	26.9±4.5	26.6±4.3	29.8±4.9	<.001
Waist circumference (cm), mean±SD	95±12.7	94±12.4	103.7±12.5	<.001
Alcohol consumption (g/day), mean±SD	12.8±16.2	13.0±16.1	11.2±16.8	0.26
Total food intake (g/day), mean±SD	1600.1±507.8	1604.4±507.4	1563.1±511.9	0.42
Total cholesterol (mg/dL), mean±SD	212.4±41.7	215.5±40.4	185.7±42.8	<.001
LDL cholesterol (mg/dL), mean±SD	129.6±37.1	132.4±36.1	106.1±37.6	<.001
HDL cholesterol (mg/dL), mean±SD	59.8±15.4	60.7±15.5	52.2±12.5	<.001
Triglycerides (mg/dL), mean±SD	115.8±57.6	113.3±56	136.7±66.0	<.001
Glucose (mg/dL), mean±SD	100.9±24.5	95.8±15.0	143.6±41.9	<.001
Insulin (pmol/L), mean±SD	50.8±31.0	48.6±29.1	69.5±39.4	<.001
HOMA-IR, mean±SD	1.9±1.4	1.7±1.2	3.5±2.0	<.001
Dyslipidemia, n (%)	477 (45.3)	406 (43.1)	71 (63.4)	<.001
Systolic BP (mmHg), mean±SD	137.7±18.6	137.2±18.5	141.9±19.2	0.01
Diastolic BP (mmHg), mean±SD	81.5±9.9	81.7±9.8	79.7±10.2	0.04
Hypertension, n (%)	530 (50.3)	449 (47.7)	81 (72.3)	<.001

BMI, Body Mass Index; BP, blood pressure; HDL, High-density Lipoprotein; HOMA-IR, Homeostasis Model Assessment of Insulin Resistance; LDL, Low-density Lipoprotein; SD, Standard Deviation; T2DM, Type 2 Diabetes Mellitus.

Study participants had a mean age of 68.6±4.5 years, 49.5% were men and 10.6% had T2DM. Compared with individuals without T2DM, individuals with T2DM showed higher frequencies of males (64.0%) and ever smokers (55.9%) and higher levels of HOMA-IR and prevalence of hypertension (p<0.001).

### 3.2 Bile acids and FGF-19

Of the 33 cBA measured through laboratory analyses, 10 cBA with more than 40% of values below LOD were considered not reliable for subsequent analyses (TCA-SULF, 7-DehydroCA, TUDCA, GHCA, TLCA-SULF, 7-KLCA, UrsoCA, GHDCA, CA-SULF, TLCA), while 23 cBA were selected (GCDCA, GUDCA-SULF, GCDCA-SULF, CDCA, Dehydro-CA, GDCA, GDCA-SULF, GUDCA, TCDCA, DCA, GCA, GLCA, CDCA-GLC, TCA, CA, DCA-GLC, CA-GLC, THDCA, LCA-SULF, THCA, TDCA, LCA, UDCA). The cBAs classification and their mean values, along with the number of samples imputed due to values under LOD or over the upper limit of the linearity range (range 0.1-37.0%), were reported in **Supplementary Table 1**. The descriptive statistics of the levels of the cBA groups by diabetes status are reported in **Table 2**. Individuals with T2DM had higher total and secondary cBAs, and lower FGF-19 levels. The descriptive statistics at the level of individual cBAs are reported in **Supplementary Table 2**.

**Table 2:** Descriptives of the levels of bile acids groups and FGF-19, overall and by diabetes status.

	All participants (n=1049)		Non-T2DM (n=938)		T2DM (n=111)		p-value^1^
	Mean±SD (log nM)	Geometric mean (nM)	Mean±SD (log nM)	Geometric mean (nM)	Mean±SD (log nM)	Geometric mean (nM)	
Total bile acids	7.8±0.6	2558.0	7.8±0.6	2521.1	8.0±0.5	2892.6	0.01
Primary bile acids	6.9±0.8	979.6	6.9±0.8	980.9	6.9±0.8	968.8	0.88
Secondary bile acids	7.3±0.5	1411.4	7.2±0.5	1380.4	7.4±0.5	1702.3	<.001
FGF-19	5.1±0.6	166.8	5.2±0.6	173.0	4.8±0.6	122.5	<.001

FGF-19, Fibroblast Growth Factor 19; SD, Standard Deviation; T2DM, Type 2 Diabetes.

^1^t-test comparing log-transformed bile acids levels between T2DM and non-T2DM individuals.

The heatmap correlation matrix among cBAs is reported in (**Supplementary Figure 1**). Low Pearson correlation coefficients were evident among cBAs within the same pathway, due to mutually exclusive processing. Primary cBAs showed a positive correlation with secondary cBAs (Spearman’s ρ=0.43, p<0.001). FGF-19 showed a positive correlation with primary cBAs (Spearman’s ρ=0.33, p<0.001) and a weaker correlation with secondary cBAs (Spearman’s ρ=0.08, p=0.01).

### 3.3 Multiple nested regression models

In the multivariable minimally-adjusted model, we observed that primary and secondary cBAs were positively associated with HOMA-IR, while FGF-19 negatively. Results remained unchanged when T2DM individuals were excluded (**Table 3**).

**Table 3:** Results of the nested linear regression models investigating the independent and mutually-adjusted associations between HOMA-IR index and bile acid groups and FGF-19.

	Minimally-adjusted model^1^			Fully-adjusted model^2^			FGF-19 - adjusted model^3^			Mutually-adjusted model^4^		
	β	95%CI	FDR-adjusted p-value^5^	β	95%CI	FDR-adjusted p-value^5^	β	95%CI	FDR-adjusted p-value^5^	β	95%CI	FDR-adjusted p-value^5^
All participants (n=1049)												
Total bile acids	0.16	0.12, 0.20	6.82×10^-14^*	0.10	0.07, 0.13	5.04×10^-08^*	0.19	0.15, 0.23	4.65×10^-19^*	nr	nr	nr
Primary bile acids	0.09	0.05, 0.13	1.77×10^-05^*	0.07	0.04, 0.11	4.52×10^-05^*	0.14	0.10, 0.18	7.65×10^-10^*	0.06	0.02, 0.10	5.23×10^-03^*
Secondary bile acids	0.18	0.14, 0.22	1.92×10^-17^*	0.09	0.06, 0.13	3.38×10^-07^*	0.19	0.15, 0.22	4.65×10^-19^*	0.07	0.04, 0.11	2.69×10^-04^*
FGF-19	-0.08	-0.12, -0.05	5.02×10^-05^*	-0.02	-0.05, 0.02	0.31	nr	nr	nr	-0.05	-0.08, -0.01	9.25×10^-03^*
Participants without T2DM (n=938)												
Total bile acids	0.14	0.10, 0.18	1.65×10^-11^*	0.09	0.06, 0.13	5.35×10^-07^*	0.18	0.14, 0.22	1.62×10^-15^*	nr	nr	nr
Primary bile acids	0.09	0.05, 0.13	2.35×10^-05^*	0.07	0.04, 0.11	5.72×10^-05^*	0.13	0.09, 0.18	1.26×10^-08^*	0.06	0.02, 0.10	8.36×10^-03^*
Secondary bile acids	0.16	0.12, 0.19	1.04×10^-13^*	0.08	0.05, 0.12	4.09×10^-06^*	0.17	0.13, 0.20	2.50×10^-15^*	0.06	0.02, 0.10	2.16×10^-03^*
FGF-19	-0.06	-0.10, -0.02	8.36×10^-03^*	0.00	-0.04, 0.03	0.84	nr	nr	nr	-0.04	-0.07, 0.00	0.05

The HOMA-IR levels were log-transformed, the FGF-19 and bile acid groups levels were log-transformed and standardized.

BMI, Body Mass Index; CI, Confidence Interval; FDR, False Discovery Rate; FGF-19, Fibroblast Growth Factor 19; HOMA-IR, Homeostasis Model Assessment of Insulin Resistance; T2DM, Type 2 Diabetes.

^1^Model 1: Adjusted for age, sex, total food intake, alcohol intake. Each bile acid group and FGF-19 is entered independently.

^2^Model 2: Model 1 + BMI, hypertension, dyslipidemia.

^3^Model 3: Model 1 + FGF-19.

^4^Adjusted for the control variables from the fully-adjusted model (Model 2), with all bile acid groups (primary and secondary) and FGF-19 entered simultaneously.

^5^Adjusted using the Benjamini-Hochberg procedure, also considering the analyses at the individual bile acid-level (**Supplementary Table S3**).

*Statistically significant after Benjamini-Hochberg correction at *q*=0.01.

The association of primary and secondary bile acids with HOMA-IR was still noticeable even in the fully-adjusted models, in all and non-T2DM cases (**Table 3**). In the respective fully-adjusted model, the association between HOMA-IR and FGF-19 was no longer statistically significant. In the FGF-19-adjusted models, the association with HOMA-IR appeared higher with secondary bile acids, but the estimate of the association with primary bile acids increased by about 50%, considering both all and non-T2DM participants. In the mutually-adjusted model, the estimates for primary and secondary cBAs became similar to each other. The strongest association was observed for the model including total bile acids instead of primary and secondary subtypes, along with FGF-19 (**Table 3**).

**Figure 2:**
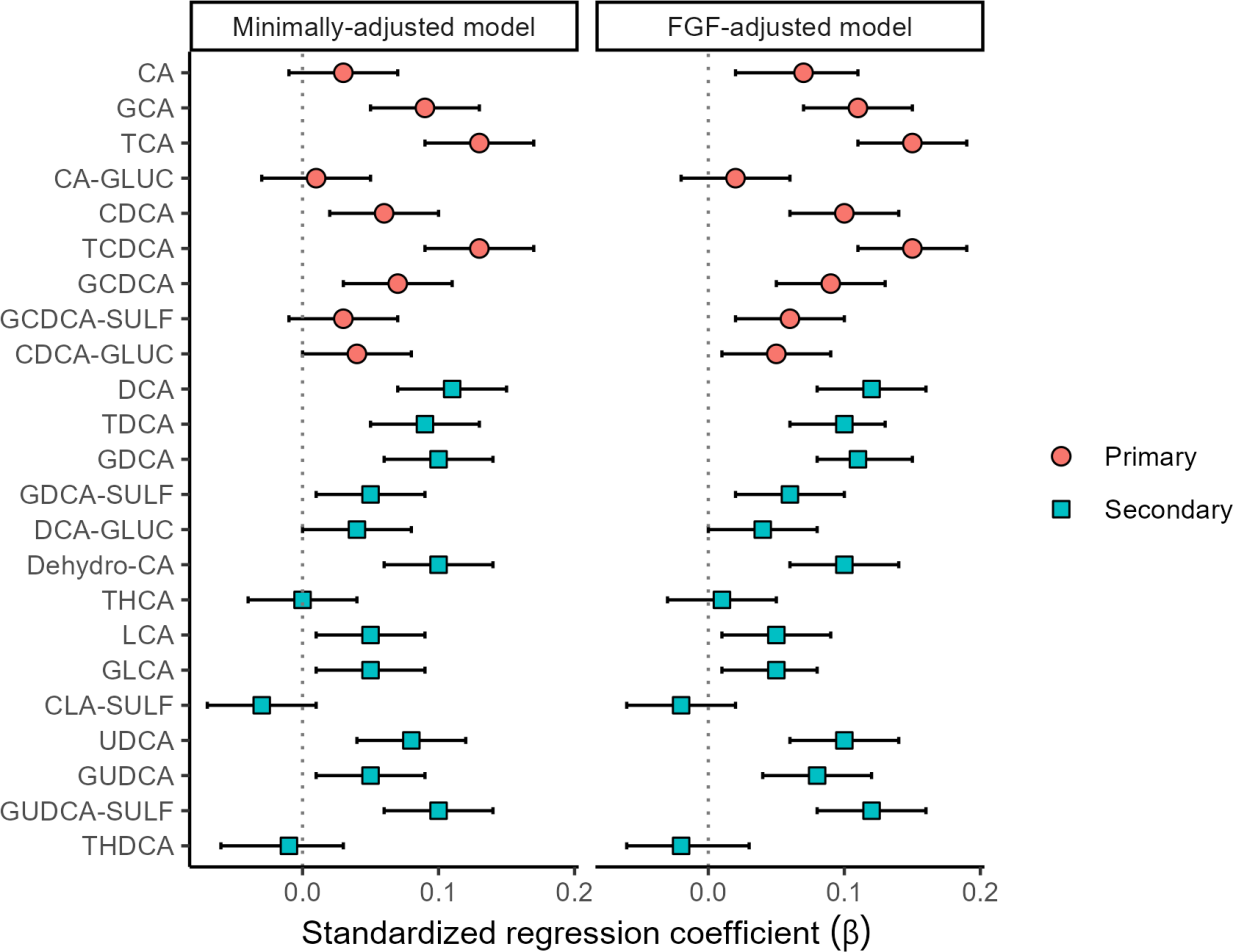
Associations (standardized regression coefficient β values and 95% confidence intervals) between HOMA-IR index and bile acids (n=1049).

When examining the association between individual cBAs and HOMA-IR, GCA, TCA, CDCA, TCDCA, GCDCA, DCA, TDCA, GDCA, Dehydro-CA, UDCA, and GUDCA-SULF were associated with HOMA-IR in the minimally- and FGF-19- adjusted models, while CA, GDCA-SULF and GUDCA in the FGF-19- adjusted models only (**Figure 2**; **Supplementary Table 3**).

**Figure 3:**
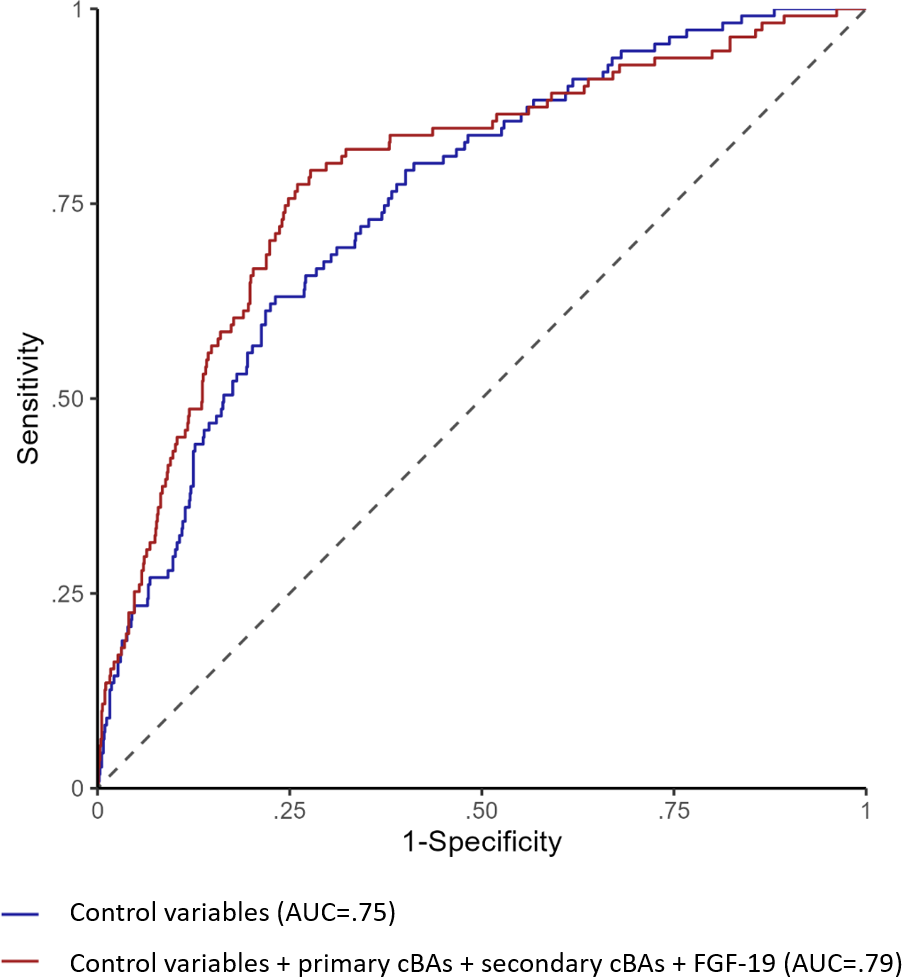
ROC curves for logistic regression models predicting type 2 diabetes. The figure shows the ROC curves for the logistic model that includes the control variables (age, sex, total food intake, alcohol intake, BMI, hypertension, dyslipidemia) in blue line and the model that also includes primary cBAs, secondary cBAs and FGF-19 in red line.

**Figure 3** shows the ROC curves for the logistic model that includes the control variables (age, sex, total food intake, alcohol intake, BMI, hypertension, dyslipidemia) and the model that also includes the cBA groups and FGF-19. The model with the cBA groups and FGF-19 showed higher ability to characterize the presence of T2DM (ΔAUC=0.03, 95% CI; 0.01-0.06). At the operating point where specificity is equal to 0.75, the model with only control variables achieved a sensitivity of 0.63, whereas the model with also the cBA groups and FGF-19 achieved a sensitivity of 0.76. Consistently, the continuous NRI was in favor of the latter model (overall NRI=0.54, 95% CI: 0.30-0.75; NRI among the events= 0.30; NRI among the non-events=0.24).

## 4 Discussion

The aim of this study was to investigate the association of HOMA-IR with fasting, circulating BA levels in participants with and without type 2 diabetes, and to evaluate the role of FGF-19 in this association, in a general population of Italian elderly from the RoCAV cohort. Finally, we investigated their role as biomarker in type 2 diabetes, to suggest a role as potential predictive markers of disease, to be further investigated in prospective studies. In this study, we identified associations with HOMA-IR index with total bile acids and FGF-19. Among the 33 cBAs identified, half of them contributed to these associations.

Circulating BAs have different chemical properties, and they can be distinguished based on their site of synthesis, either the liver (primary) and in the intestine (secondary) by the resident microbiota (20). When analyzed the relationship between cBAs and HOMA-IR index, we found an evident association, particularly with secondary cBAs, even after adjusting for age, sex, alcohol consumption, total food intake, BMI, hypertension and dyslipidemia. These results could be explained by the link between secondary bile acids and gut microbiota. A metagenome-wide association study of gut microbiota by Qin et al. (21) showed that patients with type 2 diabetes exhibit moderate gut bacterial dysbiosis compared to controls, particularly with a decline in butyrate-producing bacteria, which may be beneficial metabolically, and an increase in opportunistic pathogens. However, we observed similar associations for primary and secondary bile acids with HOMA-IR when FGF-19 levels, linked with primary cBAs, were added to the statistical model including both microbiota-generated BA subtypes. Primary BA absorbed by gut mucosa induce FGF-19, which is then secreted at basolaterally into the portal circulation (22). Results of previous studies showing lower effect for primary BA (and then suggesting a main causal role for secondary BAs) could then be influenced by unmeasured mitigating effect of protective FGF-19 levels. Therefore, fasting cBAs could be used as potential biomarkers to identify altered pathways underlying metabolic disorders, to be used for risk assessment in the general population. Moreover, as suggested by literature, the association between cBAs and altered glucose metabolism could be causal. The relationship between circulating BAs and glucose metabolism was in fact firstly observed due to the effects of pharmacological treatments like bile acid sequestrants, which are used for both dyslipidemia and diabetes management (23). Since the cross-sectional nature of this analysis, the causal effect cannot be determined and should be investigated in prospective studies.

Moreover, difference with previous studies may be explained by differences in the population, since the majority of studies were performed on Asian and American populations). Furthermore, our study was conducted in an elderly population, while most of the other studies included participants with a mean age of around 55 years.

An important role in the metabolism of bile acids is played by FGF-19, a hormone released by enterocytes following bile acid reabsorption and activation of farnesoid X receptor (FXR). FGF-19 enters the portal circulation and activates fibroblast growth factor receptor 4 (FGFR4) in hepatocytes, thereby inhibiting bile acid synthesis (5, 24). Serum FGF-19 levels follow a diurnal rhythm, peaking 90–120 minutes after the postprandial release of bile acids (24). Hence, FGF-19 released into the bloodstream after a meal appears to reduce the production of primary bile acids in preparation for the next digestive cycle. Conversely, bile acids themselves stimulate postprandial FGF-19 production, establishing a feedback regulatory loop.

In our study, circulating levels were measured after overnight fasting, which likely reflects a combination of these regulatory mechanisms. We observed a direct association between FGF-19 and circulating bile acids (cBAs), particularly stronger for primary cBAs compared to secondary ones. Assuming that we are capturing the effect of FGF-19 on the down-regulation of primary bile acid synthesis, we would have expected a similar association with secondary bile acids, given that they are derived from the primary bile acid pool. As such, we did not observe the negative feedback of FGF-19 on bile acid synthesis; instead, we observed the effect of FXR activation by primary bile acids entering the enterohepatic circulation, which subsequently induces FGF-19 production (25, 26). This suggests that, during fasting periods, the residual concentrations of cBA-dependent FGF-19 are inversely associated with HOMA-IR, independently of the negative effects of cBA. These findings may suggest a potential involvement of FGF-19 in metabolic regulation, including pathways related to BMI, hypertension, and dyslipidemia.

We found inverse association between FGF-19 and HOMA-IR index independent of cBAs. Other studies have shown that FGF-19 regulates glucose metabolism, reduces food intake, and promote weight loss (27, 28). Additionally, activation of FGF-19 leads to a reduction in gluconeogenesis and improved insulin sensitivity (29). The mechanism of FGF-19 action appears to be altered in diabetic patients, where high levels of circulation bile acids are associated with low FGF-19 levels (29). The results of this study align with existing literature (25, 27–29) on the protective role of FGF-19 in the development of type 2 diabetes. The statistical significance of the observed association between HOMA-IR and FGF-19 disappeared when BMI, hypertension and dyslipidemia were added to the statistical model, suggesting a broader association with an unhealthy metabolic status. However, when the mutually-adjusted model was applied the association with FGF-19 remained, suggesting that FGF-19 could be linked to pathways involved in the control of insulin resistance, independent of fasted circulating bile acids concentrations.

Analysis of single bile acids showed that CA, GCA, TCA, CDCA, TCDCA, GCDCA, DCA, TDCA, GCDA, GCDA-SULF, Dehydro-CA, UDCA, GUDCA, GUDCA-SULF plasma levels were positively associated with HOMA-IR index. TCDCA and TCA showed the strongest associations with HOMA-IR, and several studies showed that they were associated with HOMA-IR and correlated with an increased risk of T2DM (30, 31). Additionally, TCA, TCDCA, and TDCA were significantly related to improved glucose levels at 6 months, as observed in the study by Heianza and colleagues. Furthermore, the initial decreases in GCDCA, TCDCA, and GUDCA were also significantly associated with long-term improvements in glucose and insulin metabolism over a 2-year period (32). Moreover, we find positive associations between the primary bile acids CA and CDCA and HOMA-IR, as reported by Heianza et al, and Cairou et al. (32, 33). Parks and colleagues (25) demonstrated *in vitro* binding between bile acids and FXR, with CDCA exhibiting the strongest affinity for this receptor. However, we also found associations for DCA and UDCA, which are weaker agonists of FXR, suggesting that the observed associations could reflect the of other pathways, marked by cBA levels.

Since FGF-19 and cBAs showed negative and positive, respectively, independent associations with insulin resistance, these two factors should be considered together in future studies, to better understand the mechanisms that contribute to diabetes risk. Similarly, since our results on discriminative ability in a cross-sectional design suggests promise in marker potential, longitudinal studies are needed to confirm whether they can also serve as predictive markers for diabetes development.

### 4.1 Limitations

The limitations of the study include its cross-sectional study design, which prevents the identification of cause-and-effect relationships. In particular, while our findings suggest potential associations between cBAs, FGF-19, and indices of insulin resistance such as HOMA-IR, the study design does not allow us to establish whether these relationships are causal. Future longitudinal cohort studies or carefully designed interventional trials will be required to verify whether alterations in cBAs and FGF-19 directly influence HOMA-IR and metabolic health, or whether the associations we observed are secondary to other underlying mechanisms.

In addition, the relatively narrow age range of the study population (60 to 75 years) may restrict the generalizability of our findings to broader age groups. Moreover, we have no data on gut microbiota in our cohort. Furthermore, since samples were collected in the morning after an overnight fast, we could not assess the diurnal variation in bile acid levels and other health indices, particularly postprandial changes. Similarly, bile acid concentrations and profiles differ significantly in different body sites, intestine, liver, circulation, all of which have potential to influence BA and FGF-19 metabolism, and therefore regulation of HOMA-IR and other markers of metabolic health. However, cBAs are currently, the only realistic measure available for large cohort studies. The strengths of the study include the large sample size, the completeness and consistency of the data, and a complete set of cBA s analyzed with a sensitivity and specificity of the analytical technique employed.

## 5 Conclusion

In conclusion, in our cohort of North Italian elderly population, both total circulating BAs and FGF-19 were strongly associated with HOMA-IR, when considered in the same model. Our findings suggest that total cBAs and FGF-19 have potential as biomarkers of insulin sensitivity that should be considered for future studies investigating the mechanism underlying glucose metabolism alterations predicting Type 2 diabetes risk and its sequelae. However, prospective studies are needed to identify the specific cBA profiles responsible for a potential cause-and-effect relationship with insulin resistance and type 2 diabetes, as well as the mechanism of action of FGF-19, to explore their potential also as targets for novel preventive interventions.

## Data Availability

The original contributions presented in the study are included in the article/**Supplementary Material**. Further inquiries can be directed to the corresponding author.

## Glossary

7-dehydro-CA: 7-DehydroCholic acid
7-keto LCA: 7-Ketolithocholic acid
BMI: Body mass index
BP: Blood pressure
CA: Cholic acid
CABALA: CirculAting Bile Acids as biomarkers of metabolic health - Linking microbiotA
CBAs: Circulating bile acids
CA-GLUC: Cholic acid-Glucoronate
CA-SULF: Cholic acid-Sulfate
CDCA: ChenoDeoxyCholic acid
CDCA-GLUC: ChenoDeoxyCholic acid-glucoronate
CI: Confidence intervals
CYP7A1: Cytochrome P450 family 7 subfamily A member 1
CYP8B1: Cytochrome P450 family 8 subfamily B member 1
DCA: DeoxyCholic acid
DCA-GLUC: DeoxyCholic acid-glucoronate
Dehydro-CA: DehydroCholic acid
ELISA: Enzyme-linked immunosorbent assay
EPIC: European Prospective Investigation on Cancer and Nutrition
FGF-19: Fibroblast Growth Factor 19
FXR: Farnesoid X receptor
GCA: Glycocholic acid
GCDCA: GlycoChenoDeoxycholic acid
GCDCA-SULF: GlycoChenoDeoxycholic acid-sulfate
GDCA: GlycoDeoxycholic acid
GDCA-SULF: GlycoDeoxycholic acid-sulfate
GHCA: GlycoHyocholic acid
GHDCA: GlycohyoDecoxycholic acid
GLCA: GlycoLithocholic acid
GUDCA: GlycoUrsoDeoxycholic acid
HDL: High-density lipoprotein
HOMA-IR: Homeostatic Model Assessment for Insulin Resistance index
IR: Insulin resistance
LCA: LithoCholic acid
LCA-SULF: LithoCholic acid-Sulfate
LDL: Low-density lipoprotein
LOD: Level of Definition
MedScore: Mediterranean Diet Score
NAFLD: Non-alcoholic fatty liver disease
NRI: Net Reclassification Improvement
RCT: Randomised control trials
RoCAV: Risk of Cardiovascular diseases and abdominal aortic Aneurysm in Varese
SD: Standard deviation
T2DM: Type 2 diabetes
TCA: TauroCholic acid
TCA-SULF: TauroCholic acid-sulfate
TCDCA: TautoChenoDeoxycholic acid
TDCA: TauroDeoxycholic acid
TGR5: G-protein-coupled bile acid receptor 5
THCA: TauroHyoCholic acid
THDCA: TauroHyoDeoxycholic acid
TLCA: TauroLithocholic acid
TLCA-SULF: TauroLithocholic acid-sulfate
TUDCA: TauroUrsodeoxycholic acid
UCA: UrsoCholic acid
UDCA: UrsoDeoxyCholic acid
UHPLC-MS/MS: Ultrahigh performance liquid chromatography tandem mass spectrometry method.
