# Supplementary material for: Circulating bile acids and HOMA-IR: cross-sectional results from the RoCAV population-based study
Source: Front Endocrinol (Lausanne). 2025 Oct 15;16:1656942. doi: 10.3389/fendo.2025.1656942 (PMC12568413; doi:10.3389/fendo.2025.1656942)
Supplement: Supplementary file 1 [file DataSheet1.docx]

Supplementary Material

# Supplementary Figures and Tables

## Supplementary Figures


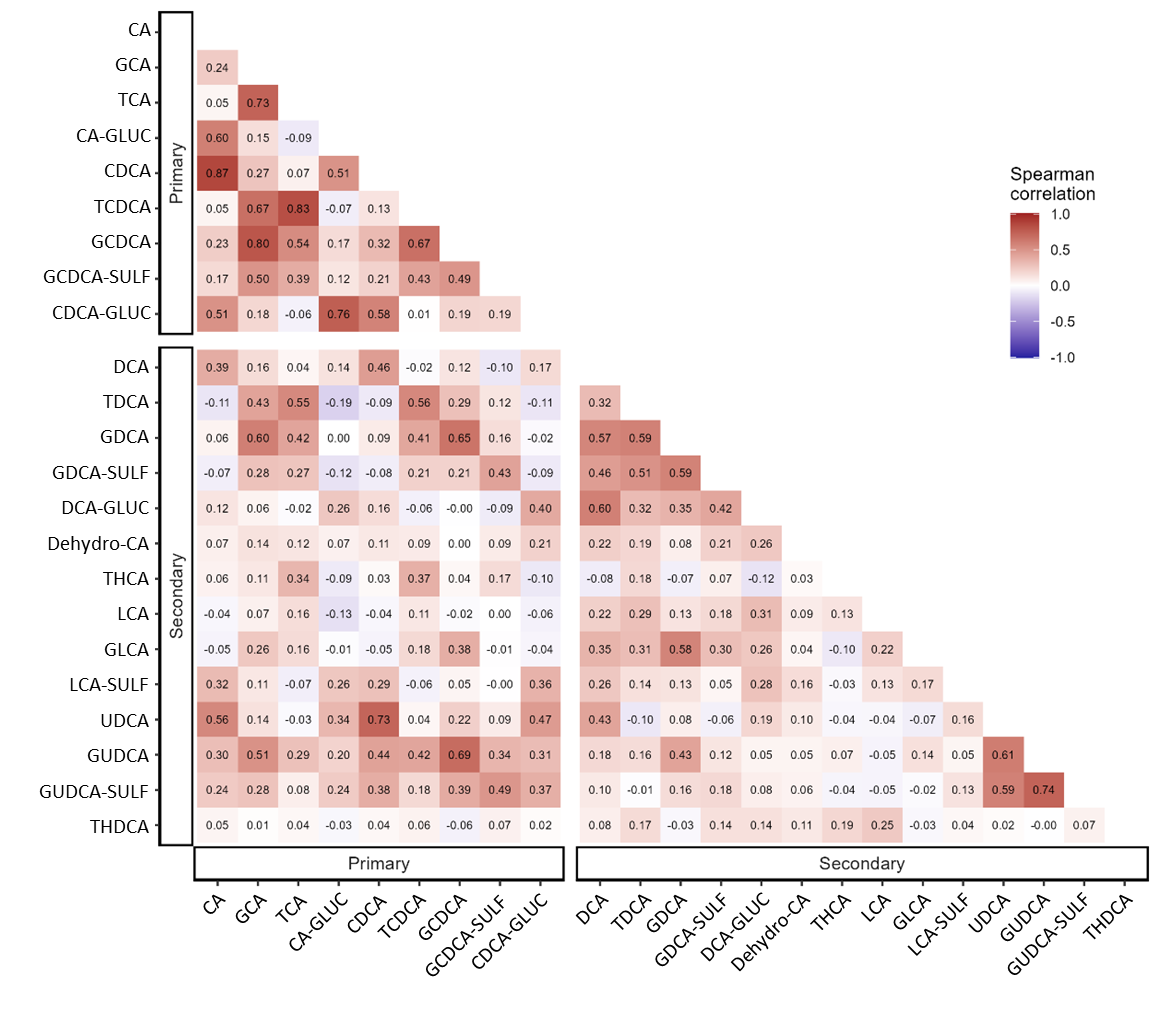


**Supplementary Figure 1.** Heatmap of the Spearman correlation matrix for 23 circulating bile acids in human plasma. Red boxes indicate the positive correlation and violet boxes the negative correlation between the two factors. Light blue boxes indicate correlation near 0.

- 1. **Supplementary Tables**

**Supplementary Table 1.** Characteristics of the bile acids measured based on High Performance Liquid Chromatography, information about limit of detection and linearity range, and information about whether the bile acid was selected for the analyses and summary statistics in the study sample.

| **Name** | **Acronym** | **Type** | **Conjugation** | **12α(OH) or non-12α(OH)** | **LOD (nM)** | **Upper limit of**  **Linearity Range**  **(nM)** | **Missing or**  **<LOD**  **(n (%))^1^** | **Selected for**  **analysis** | **N >**  **Linearity Range**  **(n (%))^1^** | **Mean ±SD**  **(nM) ^2^** | **Median [IQR]**  **(nM)** **^1^** |
| --- | --- | --- | --- | --- | --- | --- | --- | --- | --- | --- | --- |
| **Cholic acid** | CA | P | Unconjugated | 12α-hydroxylated | 4 | 6750 | 170 (16) | Yes | 0 (0) | 345.5 ±583.4 | 120 [35.9-373] |
| **Cholic acid-Sulfate** | CA-SULF | P | Unconjugated | 12α-hydroxylated | 5 | 2250 | 1079 (100) | No | nr | nr | nr |
| **Cholic acid-Glucoronate** | CA-GLUC | P | Unconjugated | 12α-hydroxylated | n\a | n\a | 211 (20) | Yes | 0 (0) | 7.4 ±9.5 | 4.7 [2.2-8.9] |
| **Glycocholic acid** | GCA | P | Glycine-conjugated | 12α-hydroxylated | 1 | 2250 | 44 (4) | Yes | 0 (0) | 83.3 ±123.6 | 44.4 [23.2-93.1] |
| **TauroCholic acid** | TCA | P | Taurine-conjugated | 12α-hydroxylated | 0.8 | 2250 | 101 (9) | Yes | 0 (0) | 19.1 ±52.4 | 7 [3.3-17.1] |
| **TauroCholic acid-sulfate** | TCA-SULF | P | Taurine-conjugated | 12α-hydroxylated | 50 | 750 | 595 (55) | No | nr | nr | nr |
| **7-DehydroCholic acid** | 7-dehydro-CA | S | Unconjugated | 12α-hydroxylated | 5 | 2250 | 838 (78) | No | nr | nr | nr |
| **DehydroCholic acid** | Dehydro-CA | S | Unconjugated | 12α-hydroxylated | 3 | 2250 | 13 (1) | Yes | 34 (3) | 792.1 ±520.4 | 644 [406-1060] |
| **DeoxyCholic acid** | DCA | S | Unconjugated | 12α-hydroxylated | 10 | 2250 | 35 (3) | Yes | 10 (1) | 357.2 ±373.3 | 247 [123-442.5] |
| **DeoxyCholic acid-glucoronate** | DCA-GLUC | S | Unconjugated | 12α-hydroxylated | n\a | n\a | 186 (17) | Yes | 0 (0) | 11.1 ±18.2 | 6.7 [3.4-12.1] |
| **UrsoCholic acid** | UCA | P | Unconjugated | non-12α-hydroxylated | 5 | 2250 | 968 (90) | No | nr | nr | nr |
| **GlycoDeoxycholic acid** | GDCA | S | Glycine-conjugated | 12α-hydroxylated | 4 | 2250 | 20 (2) | Yes | 1 (0.1) | 128 ±188.8 | 72.3 [35.7-147] |
| **GlycoDeoxycholic acid-sulfate** | GDCA-SULF | S | Glycine-conjugated | 12α-hydroxylated | n\a | n\a | 20 (2) | Yes | 0 (0) | 45.8 ±60.7 | 31.5 [15.7-55.7] |
| **TauroDeoxycholic acid** | TDCA | S | Taurine-conjugated | 12α-hydroxylated | 3 | 2250 | 323 (30) | Yes | 0 (0) | 24.5 ±36.7 | 11.9 [6.7-26] |
| **ChenoDeoxyCholic acid** | CDCA | P | Unconjugated | non-12α-hydroxylated | 3 | 2250 | 15 (1) | Yes | 39 (4) | 390.5 ±553.8 | 158.5 [57.6-451.5] |
| **ChenoDeoxyCholic acid-glucoronate** | CDCA-GLUC | P | Unconjugated | non-12α-hydroxylated | 3 | 2250 | 102 (9) | Yes | 0 (0) | 78.6 ±107.5 | 44.6 [21.1-91.3] |
| **GlycoChenoDeoxycholic acid** | GCDCA | P | Glycine-conjugated | non-12α-hydroxylated | 2.5 | 2250 | 1 (0.1) | Yes | 7 (1) | 356.4 ±365.8 | 234 [114-469] |
| **GlycoChenoDeoxycholic acid-sulfate** | GCDCA-SULF | P | Glycine-conjugated | non-12α-hydroxylated | 1 | 1100 | 3 (0.3) | Yes | 1 (0.1) | 46.3 ±60.2 | 32.3 [18.3-55.6] |
| **GlycoHyocholic acid** | GHCA | P | Glycine-conjugated | non-12α-hydroxylated | 3 | 2250 | 848 (79) | No | nr | nr | nr |
| **TauroChenoDeoxycholic acid** | TCDCA | P | Taurine-conjugated | non-12α-hydroxylated | 3 | 1500 | 27 (3) | Yes | 0 (0) | 54.6 ±93.3 | 30.5 [15.2-61.3] |
| **TauroHyoCholic acid** | THCA | P | Taurine-conjugated | non-12α-hydroxylated | 0.8 | 2250 | 326 (30) | Yes | 0 (0) | 1.3 ±0.4 | 1.2 [1.1-1.4] |
| **LithoCholic acid** | LCA | S | Unconjugated | non-12α-hydroxylated | 1 | 2250 | 361 (33) | Yes | 0 (0) | 11.6 ±9.3 | 9 [5.8-14.8] |
| **LithoCholic acid-Sulfate** | LCA-SULF | S | Unconjugated | non-12α-hydroxylated | 1.5 | 2250 | 228 (21) | Yes | 0 (0) | 11 ±11.6 | 7.7 [5.7-11.8] |
| **GlycoLithocholic acid** | GLCA | S | Glycine-conjugated | non-12α-hydroxylated | 1.3 | 2250 | 51 (5) | Yes | 0 (0) | 10.8 ±13.8 | 5.9 [3.5-12.1] |
| **TauroLithocholic acid** | TLCA | S | Taurine-conjugated | non-12α-hydroxylated | 3 | 2250 | 900 (83) | No | nr | nr | nr |
| **TauroLithocholic acid-sulfate** | TLCA-SULF | S | Taurine-conjugated | non-12α-hydroxylated | 50 | 2250 | 860 (80) | No | nr | nr | nr |
| **7-Ketolithocholic acid** | 7-keto LCA | S | Unconjugated | non-12α-hydroxylated | 9 | 2250 | 954 (88) | No | nr | nr | nr |
| **UrsoDeoxyCholic acid** | UDCA | S | Unconjugated | non-12α-hydroxylated | 18 | 2500 | 403 (37) | Yes | 0 (0) | 126.4 ±180.4 | 68.4 [36.4-144.5] |
| **GlycoUrsoDeoxycholic acid** | GUDCA | S | Glycine-conjugated | non-12α-hydroxylated | 3 | 2250 | 18 (2) | Yes | 1 (0.1) | 67.5 ±112.3 | 36.8 [16.1-77.5] |
| **GlycoUrsoDeoxycholic acid-sulfate** | GUDCA-SULF | S | Glycine-conjugated | non-12α-hydroxylated | n\a | n\a | 2 (0.2) | Yes | 0 (0) | 52.8 ±121.3 | 24.9 [12-52.8] |
| **GlycohyoDecoxycholic acid** | GHDCA | S | Glycine-conjugated | non-12α-hydroxylated | 5 | 2250 | 1067 (99) | No | nr | nr | nr |
| **TauroUrsodeoxycholic acid** | TUDCA | S | Taurine-conjugated | non-12α-hydroxylated | 3 | 2250 | 839 (78) | No | nr | nr | nr |
| **TauroHyoDeoxycholic acid** | THDCA | S | Taurine-conjugated | non-12α-hydroxylated | 6 | 2250 | 218 (20) | Yes | 4 (0.4) | 19.2 ±13.1 | 15.1 [10.2-24.4] |

Abbreviations: nr, Not Relevant; n/a, Not Available; P, Primary; S, Secondary.

^1^ relative to all participants included in the CABALA project (n=1079)

^2^ Analysis performed on participants included in the analysis of the current study (n=1049)

| **Supplementary Table 2.** Arithmetic (log-transformed) and geometric means of individual bile acids, overall and by diabetes status. | | | | | | | |
| --- | --- | --- | --- | --- | --- | --- | --- |
|  | **All (n=1049)** | | **Non-T2DM (n=938)** | | **T2DM (n=111)** | | **p-value^1^** |
|  | **Mean ±SD**  **(log nM)** | **Geometric mean (nM)** | **Mean ±SD**  **(log nM)** | **Geometric**  **mean (nM)** | **Mean ±SD**  **(log nM)** | **Geometric**  **mean (nM)** |  |
| **Primary bile acids** | | | | | | | |
| CA | 4.3 ±1.8 | 72.0 | 4.3 ±1.8 | 77.3 | 3.7 ±1.8 | 39.5 | <.001 |
| GCA | 3.7 ±1.3 | 39.8 | 3.7 ±1.3 | 39.0 | 3.9 ±1.3 | 47.8 | 0.12 |
| TCA | 1.8 ±1.3 | 6.2 | 1.8 ±1.3 | 6.0 | 2.1 ±1.4 | 8.04 | 0.04 |
| CA-GLUC | 0.4 ±2.3 | 1.5 | 0.5 ±2.2 | 1.7 | -0.3 ±2.4 | 0.75 | <.001 |
| CDCA | 5.0 ±1.5 | 145.9 | 5.0 ±1.5 | 147.7 | 4.9 ±1.4 | 132.2 | 0.43 |
| TCDCA | 3.4 ±1.0 | 29.6 | 3.4 ±1.0 | 29.0 | 3.6 ±1.1 | 35.05 | 0.09 |
| GCDCA | 5.4 ±1.0 | 228.1 | 5.4 ±1.0 | 227.3 | 5.5 ±1.0 | 235.8 | 0.72 |
| GCDCA-SULF | 3.5 ±0.8 | 31.9 | 3.5 ±0.8 | 32.2 | 3.4 ±0.8 | 29.4 | 0.27 |
| CDCA-GLUC | 3.5 ±1.4 | 32.1 | 3.5 ±1.4 | 33.2 | 3.2 ±1.4 | 24.0 | 0.02 |
| **Secondary bile acids** | | | | | | | |
| DCA | 5.3 ±1.2 | 202.0 | 5.3 ±1.2 | 195.7 | 5.6 ±1.2 | 264.2 | 0.01 |
| TDCA | 2.1 ±1.2 | 8.1 | 2.1 ±1.2 | 7.8 | 2.3 ±1.3 | 10.5 | 0.02 |
| GDCA | 4.2 ±1.2 | 67.1 | 4.2 ±1.2 | 64.9 | 4.5 ±1.1 | 90.7 | <.001 |
| GDCA-SULF | 3.3 ±1.2 | 26.1 | 3.2 ±1.2 | 25.5 | 3.5 ±1.2 | 32.3 | 0.05 |
| DCA-GLUC | 0.9 ±2.3 | 2.4 | 0.8 ±2.3 | 2.3 | 1.1 ±2.5 | 2.9 | 0.35 |
| Dehydro-CA | 6.4 ±0.9 | 585.2 | 6.3 ±0.9 | 569.9 | 6.6 ±0.9 | 732.6 | 0.01 |
| THCA | 0.0 ±0.4 | 1.0 | 0.0 ±0.4 | 1.0 | 0.0 ±0.5 | 1.0 | 0.49 |
| LCA | 1.4 ±1.3 | 3.9 | 1.3 ±1.3 | 3.8 | 1.6 ±1.4 | 5.2 | 0.02 |
| GLCA | 1.8 ±1.0 | 6.2 | 1.8 ±0.9 | 6.1 | 2.0 ±1.0 | 7.5 | 0.04 |
| LCA-SULF | 1.6 ±1.1 | 5.2 | 1.6 ±1.1 | 5.2 | 1.7 ±1.0 | 5.4 | 0.71 |
| UDCA | 3.7 ±1.1 | 39.4 | 3.7 ±1.1 | 39.8 | 3.6 ±1.1 | 36.5 | 0.45 |
| GUDCA | 3.5 ±1.1 | 34.7 | 3.6 ±1.1 | 35.1 | 3.4 ±1.2 | 31.3 | 0.33 |
| GUDCA-SULF | 3.2 ±1.2 | 25.0 | 3.2 ±1.2 | 24.8 | 3.3 ±1.2 | 26.7 | 0.53 |
| THDCA | 2.5 ±0.7 | 12.4 | 2.5 ±0.7 | 12.3 | 2.6 ±0.7 | 12.9 | 0.48 |
| Abbreviations: SD, standard deviation; T2DM, type 2 diabetes.  ^1^ t-test comparing log-transformed bile acids levels between T2DM and non-T2DM individuals | | | | | | | |

| **ESM Table 3.** Results of the nested linear regression models investigating the independent associations between HOMA-IR index and bile acid groups (n=1049). | | | | | | |
| --- | --- | --- | --- | --- | --- | --- |
|  | **Minimally-adjusted model^1^** | | | **FGF-adjusted model^2^** | | |
|  | **β** | **95%CI** | **FDR-adjusted**  **p-value^3^** | **β** | **95%CI** | **FDR-adjusted**  **p-value^3^** |
| **Primary bile acids** | | | | | | |
| CA | 0.03 | -0.01, 0.07 | 0.24 | 0.07 | 0.02, 0.11 | 4.18×10^-03^* |
| GCA | 0.09 | 0.05, 0.13 | 4.52×10^-05^* | 0.11 | 0.07, 0.15 | 5.35×10^-07^* |
| TCA | 0.13 | 0.09, 0.17 | 1.75×10^-10^* | 0.15 | 0.11, 0.19 | 8.28×10^-13^* |
| CA-GLUC | 0.01 | -0.03, 0.05 | 0.66 | 0.02 | -0.02, 0.06 | 0.27 |
| CDCA | 0.06 | 0.02, 0.1 | 4.42×10^-03^* | 0.10 | 0.06, 0.14 | 6.12×10^-06^* |
| TCDCA | 0.13 | 0.09, 0.17 | 2.04×10^-09^* | 0.15 | 0.11, 0.19 | 4.02×10^-12^* |
| GCDCA | 0.07 | 0.03, 0.11 | 1.97×10^-03^* | 0.09 | 0.05, 0.13 | 2.95×10^-05^* |
| GCDCA-SULF | 0.03 | -0.01, 0.07 | 0.14 | 0.06 | 0.02, 0.10 | 0.01 |
| CDCA-GLUC | 0.04 | 0.00, 0.08 | 0.10 | 0.05 | 0.01, 0.09 | 0.03 |
| **Secondary bile acids** | | | | | | |
| DCA | 0.11 | 0.07, 0.15 | 5.43×10^-08^* | 0.12 | 0.08, 0.16 | 3.61×10^-09^* |
| TDCA | 0.09 | 0.05, 0.13 | 3.76×10^-05^* | 0.10 | 0.06, 0.13 | 4.41×10^-06^* |
| GDCA | 0.10 | 0.06, 0.14 | 9.12×10^-07^* | 0.11 | 0.08, 0.15 | 5.04×10^-08^* |
| GDCA-SULF | 0.05 | 0.01, 0.09 | 0.02 | 0.06 | 0.02, 0.10 | 8.36×10^-03^* |
| DCA-GLUC | 0.04 | 0.00, 0.08 | 0.07 | 0.04 | 0.00, 0.08 | 0.07 |
| Dehydro-CA | 0.10 | 0.06, 0.14 | 1.44×10^-06^* | 0.10 | 0.06, 0.14 | 2.47×10^-06^* |
| THCA | 0.00 | -0.04, 0.04 | 0.90 | 0.01 | -0.03, 0.05 | 0.54 |
| LCA | 0.05 | 0.01, 0.09 | 0.01 | 0.05 | 0.01, 0.09 | 0.02 |
| GLCA | 0.05 | 0.01, 0.09 | 0.02 | 0.05 | 0.01, 0.08 | 0.03 |
| LCA-SULF | -0.03 | -0.07, 0.01 | 0.17 | -0.02 | -0.06, 0.02 | 0.31 |
| UDCA | 0.08 | 0.04, 0.12 | 9.66×10^-05^* | 0.10 | 0.06, 0.14 | 1.05×10^-06^* |
| GUDCA | 0.05 | 0.01, 0.09 | 0.01 | 0.08 | 0.04, 0.12 | 3.24×10^-04^* |
| GUDCA-SULF | 0.10 | 0.06, 0.14 | 9.86×10^-07^* | 0.12 | 0.08, 0.16 | 5.96×10^-08^* |
| THDCA | -0.01 | -0.06, 0.03 | 0.54 | -0.02 | -0.06, 0.03 | 0.49 |
| The HOMA-IR levels were log-transformed, the bile acid levels were log-transformed and standardized.  Abbreviations: BMI, Body Mass Index; CI, Confidence Interval; FDR, False Discovery Rate; HOMA-IR, Homeostasis Model Assessment of Insulin Resistance.  ^1^ Model 1: Adjusted for age, sex, total food intake, alcohol intake. Each bile acid is entered independently of the others  ^2^ Model 2: Model 1 + FGF-19  ^3^ Adjusted using the Benjamini-Hochberg procedure, also considering the analyses at the bile acid groups-level (Table 3)  * Statistically significant after Benjamini-Hochberg correction at *q*= 0.01. | | | | | | |
